# The tumor suppressor p53 connects ribosome biogenesis to cell cycle control: a double-edged sword

**DOI:** 10.18632/oncotarget.107

**Published:** 2010-05-01

**Authors:** Michael Hölzel, Kaspar Burger, Bastian Mühl, Mathias Orban, Markus Kellner, Dirk Eick

**Affiliations:** ^1^ Department of Molecular Epigenetics, Center of Integrated Protein Science (CIPSM), Helmholtz Center Munich, Munich, Germany; ^2^ current address: Netherlands Cancer Institute, Plesmanlaan 121, 1066 CX Amsterdam, Netherlands

## Abstract

Since its first description more than 30 years ago p53 has become a paradigm for a protein with versatile functions. P53 sensitizes a large variety of genetic alterations and has been entitled the guardian of the genome. Stabilization of p53 upon DNA damage is accompanied by a complex pattern of modifications, which ascertain the cellular response either in the direction of a reversible or irreversible cell cycle arrest or programmed cell death. More recently it became evident that p53 also responds to non-genotoxic cell stress, in particular if ribosome biogenesis is affected.

## P53 degradation requires ribosome biogenesis

The nucleolus is the place of ribosome biogenesis. Here, the ribosomal RNA precursor is transcribed and processed into mature 28S, 18S, and 5.8S rRNAs. Ribosomal RNAs assemble with ribosomal proteins in 40S and 60S ribosomal subunits and are exported via the nucleoplasm into the cytoplasm. In a hallmark study, Rubbi and Milner [1] identified the nucleolus as the key structure in the control of p53 stability in UV-irradiated cells. They found that localized UV-induced pyrimidine dimers in nucleoplasmic DNA failed to stabilize p53, while the same DNA damage in nucleolar DNA stabilized p53. How does DNA damage in the nucleolus differ from DNA damage in the nucleoplasm? The genes for rRNA are organized in clusters on mammalian chromosomes, and transcription of rRNA genes leads to the establishment of nucleolar structures. Thus, it was tempting to speculate that not DNA damage itself was critical for p53 stabilization, but rather impaired expression of the rRNA genes. To test this assumption, Rubbi and Milner applied chemical drugs, genetic knockdowns, or microinjection experiments with antibodies to interfere with rRNA transcription. From these studies a model emerged with ribosome biogenesis as an essential prerequisite for p53 degradation.

The production of ribosomes in the nucleolus is comparable with an assembly line in a modern car factory. Each component is delivered just in time at the right place. Already during synthesis the nascent 47S rRNA precursor associates with ribosomal and non-ribosomal proteins and assembles in mammals in the 90S pre-ribosome. The non-ribosomal proteins regulate a multitude of different steps, which involve the modification of the rRNA by methylation and pseudo-uridinylation, the removal of external and internal transcribed rRNA sequences (ETS and ITS) from the primary transcript by endo- and exonucleases, the separation of the preribosomal 90S complex into the 40S and 60S ribosomal subunits, and finally the transport of the subunits from the nucleolus into the cytoplasm. In growing cells the production of ribosomes consumes up to two-thirds of the cellular energy and the assembly line can be interrupted at many different sites.

**Fig. 1:**
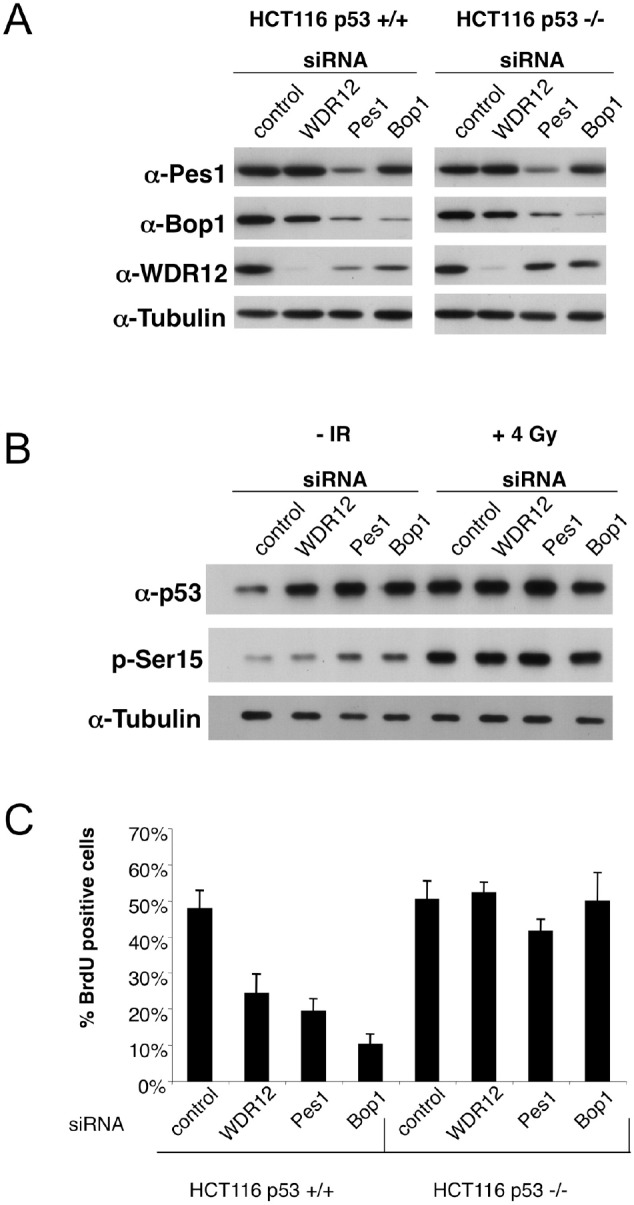
**(A)** HCT116 wild-type (p53+/+) and p53 deficient cells (p53−/−) were transfected with the indicated siRNAs. Depletion of WDR12, Pes1 and Bop1 was verified by Western blotting using the respective antibodies. Tubulin levels served as loading control. **(B)** HCT116 wild-type (p53+/+) were transfected with the indicated siRNAs. In addition, the cells were irradiated with 4 Gy before harvesting or left untreated. Levels of total and Ser15 phosphorylated p53 were determined by Western blotting. (P53 stabilization without Ser15 phosphorylation occurred also in MG132 treated cells.) **(C)** HCT116 p53+/+ and p53−/− cells were transfected with the indicated siRNAs. De novo DNA synthesis was determined by the fraction of cells with incorporation of BrdU (BrdU positive cells).

Here we have interrupted ribosome biogenesis by knockdown of three assembly factors for the 60S subunit. The factors Pes1, Bop1, and WDR12 are constituents of the PeBoW-complex. Knockdown of each component (Figure 1A) or expression of respective dominant-negative mutants strongly inhibited the maturation of 5.8S and 28S rRNA for the 60S ribosomal subunit, but did not affect maturation of the 18S rRNA for the 40S subunit [2-6]. Depletion of each factor from HCT116 cells induced a strong accumulation of p53 (Figure 1B). The same accumulation was observed, if cells were irradiated with 4 Gray (lane 5, control). Accumulation of p53 did not further increase, if the knockdown of PeBoW components and γ-irradiation were combined (lanes, 6-8). Thus, non-genotoxic inhibition of ribosome biogenesis and genotoxic γ-irradiation accomplish the same level of p53 stabilization. Notably, a significant difference in the modification of the N-terminal serine-15 in p53 was observed. Serine-15 is a target of the ATM kinase and becomes phosphorylated strongly after γ-irradiation. While knockdown of PeBoW components increased the phosphorylation of serine-15 only marginally if at all, strong serine-15 phosphorylation was detectable after γ-irradiation. Modification of serine-15 by phosphorylation inhibits the interaction of p53 with the E3 ligase Hdm2. Hdm2 controls the stability of p53 and targets p53 for proteasomal degradation by ubiquitination. Since serine-15 phosphorylation obviously plays no significant role for p53 stabilization after knockdown of Pes1, Bop1, and WDR12, other mechanisms have to be considered for p53 stabilization after inhibition of ribosome biogenesis.

## Ribosomal proteins control the stability of p53

How can the process of ribosome biogenesis control the stability of p53? If the assembly line for ribosomes is interrupted in the nucleolus, the supply with components continues and leads to an accumulation of free, non-incorporated ribosomal proteins. In fact, several ribosomal proteins have been reported to play a distinctive role in the transmission of signals for p53 stabilization. If ribosome biogenesis is disturbed, the ribosomal proteins L5, L11, and L23 associate with Hdm2. As a consequence, Hdm2 can no longer ubiquitinate and target p53 for proteasomal degradation. The involvement of L5, L11, or L23 in signaling to p53 was confirmed by knockdown experiments. The chemotherapeutic agent 5-Fluorouracil (5-FU) potently blocks rRNA processing and increases the fractions of free L5, L11, and L23. Knockdown of these ribosomal proteins by RNAi prevented p53 activation and reversed the 5-FU-induced G1/S arrest. Consistently, adding an excess of uridine to 5-FU treated cells to specifically restore the RNA metabolism also abrogated p53 accumulation [7]. These results demonstrate that 5-FU treatment triggers a ribosomal stress response so that ribosomal proteins L5, L11, and L23 are released from ribosomes to activate p53 by ablating the MDM2-p53 feedback circuit [8]. However, the association of ribosomal proteins not simply inhibited the activity of Hdm2. The association possibly alters its specificity since association of L11 with Hdm2 can trigger the ubiquitination and degradation of Hdm4/HdmX (an Hdm2 related E3 ligase that can stimulate p53 ubiquitination by Hdm2) [9]. L5, L11, and L23 are all components of the large ribosomal 60S subunit. Can subunits of the 40S small subunit also contribute to p53 stabilization and what happens, if processing of the 18S rRNA is specifically inhibited? This was achieved either by knockdown of the ribosomal S6 or S7 proteins [10-12], or by knockdown of the 18S rRNA specific processing factor UTP18 [13]. In all instances p53 became stabilized and unexpectedly, this stabilization required the presence of L11 protein of the large subunit in mammalian cell culture cells [11, 13]. Therefore, cells survey the maturation of the small and large ribosomal subunits by separate molecular routes, which may merge in an L11-dependent signaling pathway for p53 stabilization. Interestingly, loss of L11 affects zebrafish embryonic development through a p53-dependent apoptotic response [14] suggesting that defects in ribosome biogenesis can activate p53 by L11-dependent and independent pathways.

## The bright side of p53

The dominance of p53 in the literature correlates well with its mutation rates in cancer. P53 mutations occur at all stages of tumor development and all major forms of cancers benefit from p53 mutations. The loss of p53 removes a control mechanism from cells that recognizes cellular damage in a sophisticated manner and orchestrates the appropriate response. P53 either arrests a damaged cell in the division cycle or induces its elimination by programmed cell death (apoptosis). Both biological activities of p53 have been intensively studied in the past and have been confirmed in large panels of different mouse tumor models. Notably, mice with an increased copy number of wild-type p53 (super p53 mice) were significantly protected from cancer when compared with normal mice [15] further supporting p53's outstanding role in the control of tumor development. This bright side of wild-type p53, the prevention of cancer, has been reviewed in a large number of publications in the past. For recent reviews see [16, 17]. Here we will focus on the dark side of wild-type p53.

## The dark side of p53

Unfortunately, wild-type p53 also possesses a dark side that contributes to the development of various diseases and syndromes. Interestingly, this dark side is tightly connected with defects in ribosome biogenesis. During the recent years many genes encoding ribosomal proteins have been identified as the origin of congenital human disorders. The affected genes encode for ribosomal proteins of the 40S as well as 60S subunits. A prominent example is Diamond Blackfan anemia (DBA), a congenital bone marrow failure syndrome of the erythroid lineage. DBA is characterized by macrocytic anemia, congenital anomalies and a predisposition to cancer. The genes mutated in DBA encode ribosomal proteins associated with the 40S as well as 60S subunit formation. The mutated genes identified so far include S7, S17, S19, and S24 of the small ribosomal subunit, and L5, L11, and L35a of the large subunit (for details of mutations see the Diamond-Blackfan anemia database, www. dbagenes.unito.it; [18]). Until now this database describes 148 disease-associated variants of ribosomal genes with the highest frequency of variants for S19 (83), followed by L5 (29) and L11 (23), and a lower frequency for S24 (6), L35a (4), S17 (2), and S7 (1). More recently, a second ribosome-biogenesis associated disease was identified. The 5q-syndrome is a subtype of the myelodysplastic syndromes (MDS) with loss of a distinctive region on chromosome 5q. It is characterized by a defect in erythroid differentiation, macrocytic anemia and the risk of transformation to acute myeloid leukemia (AML). An RNAi-based approach could recently discover the ribosomal S14 gene as the 5q- disease gene [19, 20]. Similar as in DBA-cells, the haploinsufficiency of a ribosomal gene caused an rRNA processing defect in 5q- cells, which was rescued by reconstitution of S14. A recent study indicated that S14 expression is also affected in cells of a large portion of MDS patients without 5q- deletion [21] suggesting that, besides other possible ribosomal genes, S14 is primarily affected in MDS.

In addition to DBA and MDS, abnormalities in ribosomal gene regulation or ribosome biogenesis are the basis for further genetic diseases, including Shwachman-Diamond syndrome [22, 23], dyskeratosis congenita [24], or cartilage-hair hypoplasia [25]. Mouse models have been established for DBA and MDS, which largely mimic the human diseases. As for DBA, mutations in S19 resulted in reduced body size and erythrocyte count accompanied by an accumulation of p53 [26]. Haploinsufficency of S14 in MDS mice caused macrocytic anemia, prominent erythroid dysplasia and monolobulated megakaryocytes in the bone marrow [27]. Both transgenic mouse models suffered from defective bone marrow progenitor development, the appearance of bone marrow cells expressing high amounts of p53 and increased bone marrow cell apoptosis. Intercrossing the DBA and MDS mice with p53-deficient mice completely rescued the progenitor cell defect and restored a normal hematopoietic stem cell bone marrow population. These mouse models suggest that a p53-dependent mechanism underlies the pathophysiology of DBA and MDS. But why have these diseases also a high incidence for tumor development? Are high levels of p53 in bone marrow cells of DBA and MDS patients the basis for selection of cells with further genetic lesions, which counteract the high apoptosis rate? Do new lesions affect even p53 itself? These open questions will be answered probably in the next future.

## CONCLUSIONS

Ribosome biogenesis in mammalian cells, previously regarded only as a process for the production of ribosomes, comes now into focus of pathophysiological mechanisms. Mutant genes for ribosomal proteins are increasingly identified as the basis for human diseases with p53 as possible downstream effector. In cancer treatment, p53 is also an important effector for the efficacy of chemotherapeutic drugs. The molecular mechanisms of p53 activation in tumor cells by chemotherapy are often not clarified and a possible contribution of inhibition of ribosome biogenesis has not been investigated. Therefore we recently analyzed the impact of chemotherapeutic drugs on ribosome biogenesis [28]. Notably, 19/36 substances inhibited ribosome biogenesis at the level of rRNA transcription or rRNA processing. Thus, inhibition of ribosome biogenesis by chemotherapy could be an important aspect of p53 activation in tumor cells and therapy success.
